# Oxidative stress-induced cellular senescence desensitizes cell growth and migration of vascular smooth muscle cells through down-regulation of platelet-derived growth factor receptor-beta

**DOI:** 10.18632/aging.102270

**Published:** 2019-10-03

**Authors:** Chun-Hsu Pan, Chang-Jui Chen, Chun-Ming Shih, Ming-Fu Wang, Jie-Yu Wang, Chieh-Hsi Wu

**Affiliations:** 1 School of Pharmacy, Taipei Medical University, Taipei 11031, Taiwan; 2 Department of Pharmacy, Taipei Medical University Hospital, Taipei 11031, Taiwan; 3 Division of Cardiology, Department of Internal Medicine, Taipei Medical University Hospital, Taipei 11031, Taiwan; 4 Department of Food and Nutrition, Providence University, Taichung 433, Taiwan

**Keywords:** aging, cellular senescence, platelet-derived growth factor, restenosis, vascular smooth muscle cells

## Abstract

The relationship between aging and restenosis are unclear. The purposes of this study were to investigate the possible pathological role and mechanism of aging on formation of restenosis. Our data indicated that cell proliferation and migration of the oxidative stress-induced senescent vascular smooth muscle cells were obviously desensitized to stimulation by platelet-derived growth factor (PDGF)-BB, which may have been caused by suppression of promoter activity, transcription, translation, and activation levels of PDGF receptor (PDGFR)-β. The analyzed data obtained from the binding array of transcription factors (TFs) showed that binding levels of eighteen TFs on the PDGFR-β promoter region (-523 to -1) were significantly lower in senescent cells compared to those of non-senescent cells. Among these TFs, the bioinformatics prediction suggested that the putative binding sites of ten TFs were found in this promoter region. Of these, transcriptional levels of seven TFs were markedly reduced in senescent cells. The clinical data showed that the proportion of restenosis was relatively lower in the older group than that in the younger group. Our study results suggested that a PDGFR-β-mediated pathway was suppressed in aging cells, and our clinical data showed that age and the vascular status were slightly negatively correlated in overall participants.

## INTRODUCTION

The proportion of the population that is aging has been increasing yearly worldwide, which is mainly attributed to improvements in healthcare and reductions in fertility rates. Hence, research on preventive medicine for a pathophysiological understanding of geriatric diseases has gradually been taken more seriously for disease prevention and health promotion of older populations. Physiological aging can be considered a natural process of functional decline or disorder from molecular to systemic levels. The free radical theory originally proposed by Denham Harman is one of the famous theories to explain possible causes of aging [1, 2]. Intracellular reactive oxygen species (ROS) or free radicals are mainly produced by nicotinamide adenine dinucleotide phosphate (NADPH) oxidase or leak from the mitochondrial electron transport chain. The progressive accumulation of ROS can induce oxidative stress and damage normal functions of macromolecules, such as nucleic acids, proteins, and lipids, resulting in some detrimental effects, e.g., mutations of nucleic acids, deactivation or destruction of proteins, and lipid peroxidation. Cobalt dichloride (CoCl_2_) was found to promote oxidative stress by producing free radicals *in vivo* and *in vitro* as well as mimicking chemical hypoxia by preventing the degradation of the intrinsic hypoxia marker, hypoxia-inducible factor (HIF)-1α [3–5]. ROS-mediated DNA damage and signaling pathways were proven to be associated with damage-induced cellular senescence [6]. Accordingly, cobalt dichloride is used as a hypoxia-mimicking agent to create cellular hypoxia and senescence (aging) by promoting the dramatic accumulation of intracellular ROS levels.

Some cardiovascular diseases, such as atherosclerosis, myocardial hypertrophy, and hypertension, are positively associated with aging progression, but the aging effect on restenosis prevalence is unclear. Restenosis (or neointimal hyperplasia) is a common complication after the surgical interventions of balloon angioplasty and vascular stenting, the pathological mechanisms of which were shown to be involved in abnormal proliferation and migration of vascular smooth muscle cells (VSMCs). The critical roles of platelet-derived growth factor (PDGF) and its receptor (PDGF receptor-β; PDGFR-β) have been well studied in VSMC proliferation and migration as well as the development of restenosis [7–9]. Therefore, we attempted to investigate differences between normal and senescent VSMCs in terms of PDGF-stimulated cell proliferation and migration, as well as to clarify whether these responses are associated with the regulation of PDGFR-β. In addition, a clinical prospective study was also conducted to determine if a relationship exists between aging and the prevalence of restenosis.

## RESULTS

### Cobalt dichloride induced VSMC senescence

**Figure 1:**
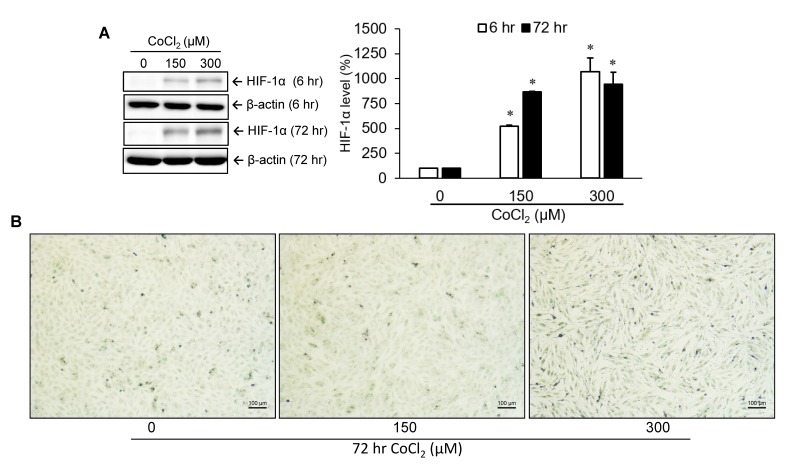
**Identification of cellular senescence.** A10 cells were incubated with various doses of cobalt dichloride (CoCl_2_) for 6 and 72 hr. The protein level of hypoxia-inducible factor (HIF)-1α was analyzed using Western blotting (**A**). Senescent cells were detected by cytochemical staining of SA-β-gal activity which appeared as a blue-green color (**B**). * *p* < 0.05 compared to the group without CoCl_2_ treatment.

HIF-1α, a critical transcription factor involved in hypoxia-mediated gene expression, was markedly upregulated at an early time point (6 hr) after treatment with several concentrations (0, 150, and 300 μM) of cobalt dichloride. An increased level of HIF-1α could still be detected at 72 hr post-treatment (Figure 1A). To identify the production of cellular senescence, senescence-associated β-galactosidase (SA-β-gal) activity within cells was detected by cytochemical staining (Figure 1B). Experimental results revealed that 72 hr of treatment with cobalt dichloride dose-dependently induced blue-green staining of a portion of cells, a representative feature of senescent cells. This result suggested that cell senescence was promoted under incubation condition with 300 μM CoCl_2_ for 72 hr.

### Cell proliferation and migration were reduced in senescent VSMCs

**Figure 2:**
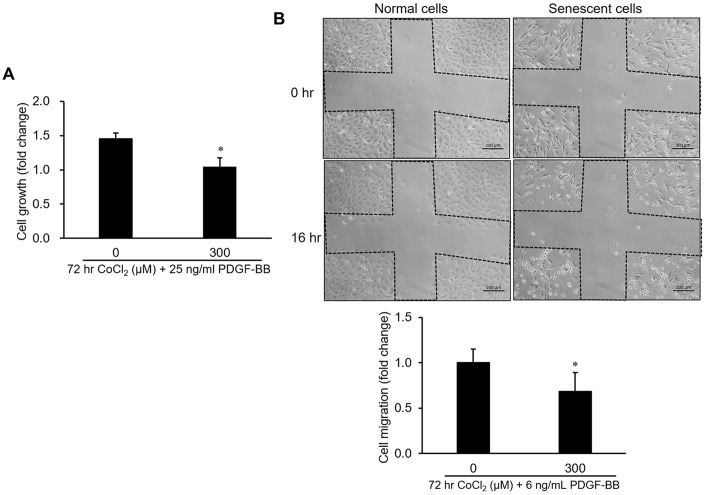
**Influence of cellular senescence on the proliferative and migratory capacities of A10 cells.** Senescence was produced in cells by a 72-hr incubation with 300 μM CoCl_2_. After that, platelet-derived growth factor (PDGF)-BB-stimulated cell proliferation and migration were respectively analyzed by an MTT assay (**A**) and wound-healing analysis (**B**). * *p* < 0.05 compared to the normal cell group.

To understand the influence of CoCl_2_-induced cellular senescence on VSMC proliferation and migration, both normal and senescent cells were incubated with PDGF-BB for 24 hr. Data from the cell growth analysis suggested that the growth capacity of senescent cells had obviously decreased compared to normal cells (Figure 2A). Likewise, cell migration of senescent VSMCs was insensitive to PDGF-BB-mediated stimulation according to data from the wound-healing assay (Figure 2B).

### Expression and activation levels of PDGFR-β and its downstream signaling molecules were suppressed in senescent VSMCs

**Figure 3:**
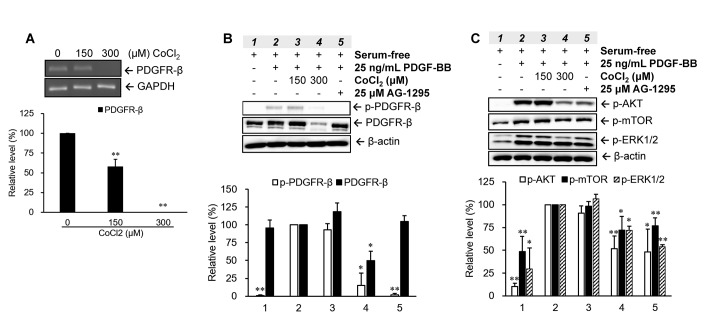
**Changes in platelet-derived growth factor (PDGF) receptor (PDGFR)-β-mediated pathways in senescent vascular smooth muscle cells (VSMCs).** Cells were treated with different concentrations of CoCl_2_ for 72 hr. Expression and activation levels of PDGFR-β were analyzed using an RT-PCR (**A**) and Western blotting (**B**), respectively. The phosphorylation levels of the downstream signaling molecules of PDGFR-β were examined after 15 min of stimulation with 25 ng/ml PDGF-BB under serum-free condition (**C**). * *p* < 0.05, ** *p* < 0.01 compared to the group treated with 10% FBS or PDGF-BB alone.

Regulations of the PDGFR-β gene and protein were analyzed in VSMCs treated with various concentrations (0, 150, and 300 μM) of CoCl_2_ for 72 hr. Our data indicated that CoCl_2_ treatment diminished gene and protein expressions of PDGFR-β in a dose-dependent manner (Figure 3A, 3B). Moreover, the phenomenon of PDGFR-β activation (phosphorylation) induced by 25 ng/ml of PDGF-BB was also obviously attenuated in VSMCs treated with 300 μM CoCl_2_, the repression of which was very similar to the inhibitory effect of AG-1295, a PDGFR blocker (Figure 3B). Likewise, activation levels of downstream signaling molecules of PDGFR-β, such as AKT, mTOR, and ERK1/2, were increased after PDGF-BB incubation, which was reversed by adding 25 μM of AG-1295 to normal cells. However, the PDGF-stimulated increases in the activations of AKT, mTOR, and ERK1/2 proteins were obviously reduced in cells incubated with 300 μM CoCl_2_ for 72 hr (Figure 3C).

### Promoter activity of PDGFR-β was reduced in senescent VSMCs

**Figure 4:**
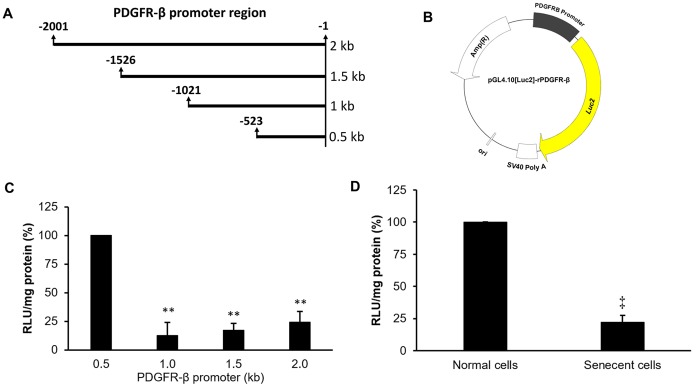
**Promoter deletion assay of platelet-derived growth factor receptor (PDGFR)-β.** Four lengths of the PDGFR-β promoter (**A**) were individually constructed in a luciferase-based reporter vector to produce four pGL4.10[*Luc2*]-rPDGFR-β vectors (**B**). Individual promoter activities were measured in normal cells at 24 hr after transfection with the different pGL4.10[*Luc2*]-rPDGFR-β plasmids (**C**). Differences in promoter activities between normal and senescent cells with the PDGFR-β segment (0.5 kb) were measured (**D**). The transcription start site was defined as +1. ** *p* < 0.01 compared to the group transfected with the reporter vector with the proximal promoter (0.5 kb) of PDGFR-β. ^‡^ *p* < 0.01 compared to the normal cell group.

To further understand the mechanism of downregulation of the PDGFR-β gene in senescent cells, several lengths (0.5, 1.0, 1.5, and 2.0 kb) of PDGFR-β promoter segments were cloned into a luciferase-based reporter vector, pGL4.10[*Luc*2], to measure their individual promoter activities (Figure 4A, 4B). The analyzed data indicated that the proximal segment ranging -523 to -1 of the PDGFR-β promoter possessed the highest promoter activity among all promoter segments (Figure 4C). Therefore, the constructed reporter vector with the proximal segment (-523 to -1) of the PDGFR-β promoter was further transfected into both normal and senescent cells, and the experimental data demonstrated that promoter activity was significantly reduced in senescent cells compared to that of normal cells (Figure 4D).

### TF-binding profile analysis of the rat PDGFR-β promoter

To evaluate differences in the binding profiles of 96 TFs on the proximal segment of the PDGFR-β promoter between normal and senescent cells, nuclear extracts isolated from normal or senescent cells were incubated with the proximal segment of the PDGFR-β promoter and a fluorescence-labeled competitive oligonucleotide probe to identify possible TF-DNA interactions using a commercial TF-DNA-binding assay kit. Our results showed that binding levels of 18 TFs obviously decreased (>2-fold difference) in senescent cells compared to those of normal cells (Table 1). Of these, binding levels of four TFs, including NFAT, SATB1, FOXD3, and PPAR, were more than 10-fold lower in senescent cells than that in normal cells.

**Table 1:** Transcription factor-binding profile analysis of the rat platelet-derived growth factor receptor-β promoter (523 bp).

**No.**	**Transcription factor**	**Binding ratio of normal cells to senescent cells**
1	NFAT (nuclear factor of activated T-cells)	29.6
2	SATB1 (special AT-rich sequence-binding protein 1)	19.4
3	FOXD3 (Forkhead box D3)	19.0
4	PPAR (peroxisome proliferator-activated receptor)	17.4
5	MEF1 (myocyte enhancer factor 1)	8.9
6	SMUC (Snail-related transcription factor Smuc)	8.4
7	HOX4C (Homeobox 4C)	7.2
8	TFIID (TATA box binding protein)	6.6
9	TCF/LEF (Runt-related transcription factor 2)	5.1
10	NRF1 (nuclear respiratory factor 1)	5.0
11	COUP-TF (nuclear receptor subfamily 2, group F)	3.9
12	PXR (pregnane X receptor)	3.9
13	OCT4 (POU class 5 homeobox 1)	3.7
14	Stat5 (signal transducer and activator of transcription 5)	3.5
15	GR/PR (glucocorticoid receptor/progesterone receptor)	3.4
16	USF-1 (upstream transcription factor 1)	3.4
17	FOXG1 (FOXbox G1)	2.5
18	Pbx1 (pre-B cell leukemia transcription factor-1)	2.1

### Bioinformatics prediction of potential TFBSs on the PDGFR-β promoter

**Figure 5:**
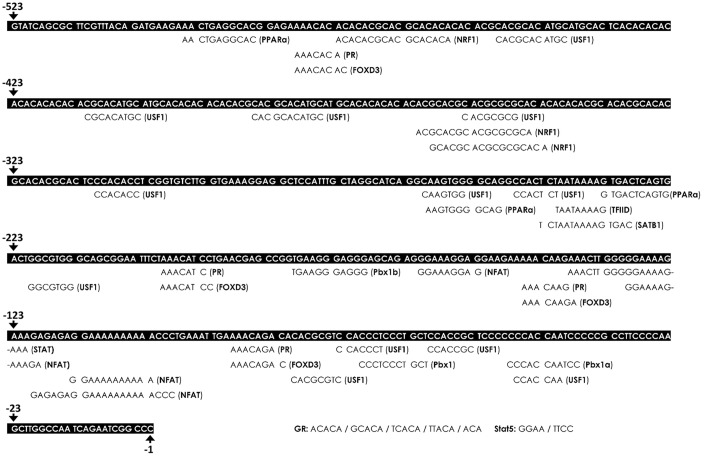
**Bioinformatics prediction of putative transcription factor-binding sites (TFBSs) on the platelet-derived growth factor receptor (PDGFR)-β promoter.** The putative TFBSs were analyzed on the PDGFR-β promoter region (-529 to -1). The sequence of the PDGFR-β promoter is presented as white text on a black background. The locations of the predicted TFBS are labeled below the PDGFR-β promoter sequence. Transcriptional start site was defined as the +1 position.

To investigate whether these 18 TFs can possibly interact with the proximal segment (-523 to -1) of the PDGFR-β promoter, potential binding sites of these TFs were analyzed and predicted by two TRANSFAC^®^-based bioinformatics on-line software programs (PROMO and MatInspector). According to the prediction results, putative binding sites of the TFs, including NFAT, SATB1, FOXD3, PPAR, TFIID, NRF1, Stat5, GR/PR, USF-1, and Pbx1, were predicted to be within the proximal segment (-523 to -1) of the PDGFR-β promoter (Figure 5).

### Gene expressions of candidate TFs in senescent VSMCs

**Figure 6:**
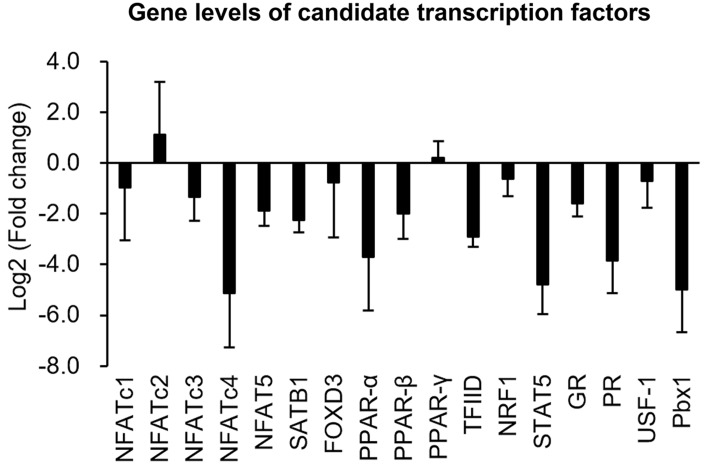
**Quantifications of gene levels of candidate transcription factors.** Transcriptional expressions of candidate transcription factors in normal and senescent cells were analyzed by a real-time PCR. Gene expression is shown as the log2 (multiple of change) of the transcription factors after senescence induction.

To clarify whether decreases in TF-binding levels could be attributed to reductions in TF expressions in senescent cells, transcriptional levels of these ten TFs were further measured using a quantitative polymerase chain reaction (qPCR). The experimental data showed that gene expressions of seven TFs, viz., NFATc4, SATB1, PPAR-α, TFIID, PR, STAT5, and Pbx1, were markedly reduced (>2-fold change) in senescent cells compared to those in normal cells (Figure 6).

### Analysis of the relationship between age and in-stent restenosis

To understand possible relationships between age and in-stent restenosis, clinical records of coronary artery disease patients with vascular stenting were collected and divided into younger (<45 years old; *n*=8) and older (>60 years old; *n*=36) groups. The data indicated that proportions of restenosis were 75.0% and 55.6% in the younger and older groups, respectively (Table 2). Moreover, younger men with vascular stenting had a relatively higher proportion of restenosis compared to that in older men (75.0% vs. 60.0%). In the older group, the proportion of restenosis in men was relatively higher than that in women (60.0% vs. 45.5%). However, Pearson’s correlation test showed that no significant correlation (*r* = -0.057, *p* = 0.725) existed between age and the vascular status (with or without restenosis) in overall patients who had been followed-up 6 months after vascular stenting. Similarly, there was also no statistical correlation when analyzing different gender groups (*r* = -0.064, *p* = 0.735 for males; *r* = 0.176, *p* = 0.606 for females).

**Table 2:** Demographic data of coronary artery disease patients with vascular stenting.

	**Younger (<45 years old)**	**Older (>60 years old)**
**Total participants**	8	36
Male	8	25
Female	0	11
**Average age (years)**	39.8 ± 4.0	73.3 ± 8.3
**Proportion with restenosis (%)**	6/8 (75.0%)	20/36 (55.6%)
Male (%)	6/8 (75.0%)	15/25 (60.0%)
Female (%)	0/0 (-)	5/11 (45.5%)

* Vascular status (with or without restenosis) of patients was diagnosed at the first follow-up visit, and the follow-up duration of patients was about 6 months.

## DISCUSSION

Accumulating ROS, important regulators in aging progression, have been reported in aged arteries. In human carotid specimens, higher ROS levels were found in the older age group (>70 years old) [10]. Similarly, results obtained from rodent studies also showed that arterial oxidative stress was significantly higher in aged groups [11–13]. Accumulating ROS in aged arteries may contribute to mitochondrial dysfunction as well as imbalances in expressions or activities of oxidant/antioxidant enzymes, e.g., NADPH oxidase, nitric oxide synthase (NOS), superoxide dismutase (SOD), and catalase (CAT). For example, NADPH oxidase activity was upregulated in carotid arteries of older patients (>70 years old), but no significant difference in SOD or CAT activities was detected between older and younger groups [10]. Moreover, upregulation of endothelial NOS expression and activity, accumulation of superoxide and protein nitrotyrosination, as well as a reduction in SOD activity were also obviously observed in the aortas of aged rodents [11–13]. In addition, it was documented that positive regulatory mechanisms of ROS on cellular senescence, a crucial event of aging, can be carried out by different regulatory routes including mitochondrial DNA damage, autophagy, microRNA induction, specific signaling pathways, etc [14]. Hence, CoCl_2_ was applied in the present study to promote accumulation of intracellular ROS and subsequent cellular senescence for mimicking a diseased situation of VSMCs within aging arteries.

Previous studies observed that cultured human arterial VSMCs harvested from the aorta of older donors had a lower growth capacity than those from younger donors [15, 16]. Likewise, age-related inhibition in wall damage-induced cell proliferation of VSMCs was also confirmed in aortas of aged rabbits [17]. It has been found that the enzymatic activity of senescence-associated beta-galactosidase, a specific marker in senescent cells, was significantly increased in primary aortic VMSCs isolated from old Brown Norway rats, which indicated VSMCs exhibited senescent phenotype in aged arterial tissues [18]. The occurrence of cellular senescence in aged arteries can explain part of the reasons in decrease of growth capacity of VSMCs. Our study demonstrated that VSMCs with CoCl_2_-induced senescence were desensitized to PDGF-BB-stimulated cell proliferation (Figure 2A). Additionally, our clinical data also found that although the older group (>60 years old) had a higher prevalence of atherosclerosis, the prevalence of restenosis was lower in older group than that of the younger group (Table 2). The pathological mechanisms of a lower restenosis prevalence in the aged group may partly be attributed to downregulation and deactivation of PDGFR-β (Figure 3) as well as consequent desensitization to PDGF-BB-mediated cell growth and migration in senescent VMSCs (Figure 2). Similarly, other studies also demonstrated that a significant reduction in balloon injury-induced neointimal hyperplasia was found in aged Wistar rats (18~27 months) [19, 20]. Nevertheless, some researchers also provided opposite evidences that the aging group had a higher prevalence of restenosis. Vazquez-Padron et al. mentioned that aging exacerbated wire injury-induced neointimal formation and increased VSMC proliferation in aged female C57BL/6 mice (18 months) [21]. Increased neointimal thickening in the carotid artery after balloon angioplasty was also found in aged Fischer 344 rats (22~24 months) [22–24]. These controversial results of an aging effect on the restenosis prevalence may have been caused by differences in patient characteristics, such as race, gender, selection criteria (e.g., comorbidity status), surgical interventions (e.g., balloon angioplasty vs. vascular stenting), medicinal interventions, and lifestyle factors (e.g., smoking).

**Figure 7:**
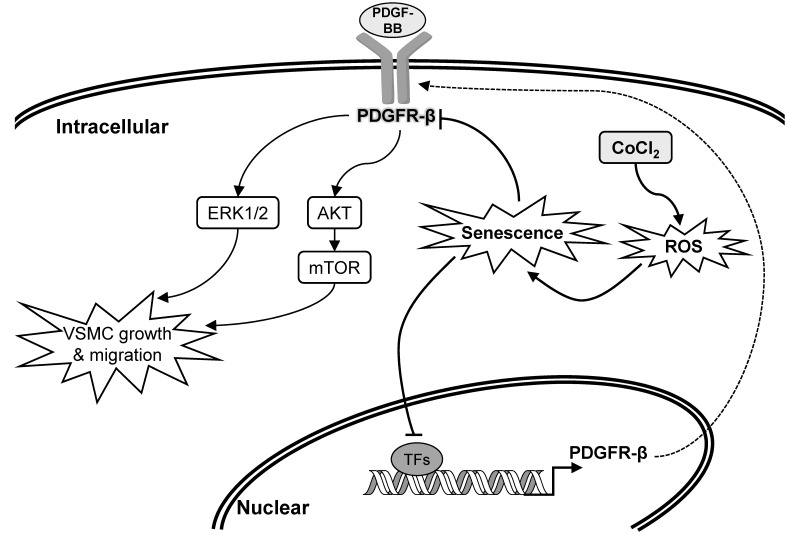
**Schematic overview of the possible pathological role and influence of vascular smooth muscle cell (VSMC) senescence on neointimal hyperplasia (restenosis).**

In the present study, a high-throughput binding array of TFs was used to recognize potential interactions between candidate TFs and the PDGFR-β promoter region (-523 to -1) in normal VSMCs and those with CoCl_2_-induced senescence. Our analytical results suggested that binding levels of 18 TFs were significantly reduced in senescent VSMCs (Table 1). After further assessment by a bioinformatics analysis and gene quantification, putative binding sites of seven TFs were found to be in the analyzed region (-523 to -1) of the PDGFR-β promoter, and gene levels of these TFs were obviously reduced in CoCl_2_-induced senescent cells (Figures 6, 7). Of these, some TFs can be modulated by oxidative stress and play critical roles in cellular senescence as well as cell growth and migration. NFAT is a calcium-dependent TF involved in phenotypic modulation of VSMCs, which was demonstrated to promote VSMC motility by some agonists (e.g., PDGF-BB and thrombin) of receptor tyrosine kinases or G protein-couple receptors [25, 26]. Use of the NFAT inhibitor, A-285222, was demonstrated to decrease serum-induced cell proliferation of cultured VSMCs [27]. Peroxisome proliferator-activated receptor (PPAR)-α, a redox-sensitive TF, is involved in lipid metabolism, and its agonists, such as fibrates, have been used in the clinic to reduce hypertriglyceridemia. Moreover, clofibrate, a PPAR-α agonist, was also confirmed to protect the heart from myocardial ischemia-induced oxidative damage by increasing expressions and activities of antioxidant enzymes, including SOD and CAT, as well as by decreasing expressions of angiotensin (Ang) II and the Ang II type 1 receptor [28]. In addition, the PPAR-α protein was expressed by various cardiovascular cells (e.g., human aortic smooth muscle cells and endothelial cells) and was found to participate in balloon angioplasty-induced restenosis [29]. Interestingly, transcription levels of PPAR-α were found to be reduced in aged rodents [30, 31]. Lena et al. mentioned that microRNA 191 can directly target the 3’ untranslated region of SATB1 mRNA to trigger keratinocyte senescence, which shows a possible role of SATB1 in the process of cellular senescence [32]. TFIID consists of the TATA-box-binding protein (TBP) and TBP-associated factors (TAFs), which form a pre-initiation complex to enhance the recruitment of RNA polymerase II and subsequent gene transcription. In VSMCs, an oxidative stress-mediated increase in the binding of p53 to the TBP was shown to reduce associations of TBP-DNA and thus suppress gene transcription [33]. In leukemic and hematopoietic cells, STAT5 promoted cell proliferation and differentiation as well as antiapoptotic activities [34, 35]. Similarly, STAT5 also plays a critical role in promoting thrombin-mediated VSMC growth and motility [36]. STAT5 signaling has been applied as a therapeutic target for cancer therapy [37]. It was revealed that Pbx1 provides a beneficial effect of protecting against oxidative stress by increasing Nfe2l1 (also called NRF1), and decreasing levels of both TFs were found in midbrain dopaminergic neurons of Parkinson's patients [38]. Pathological mechanisms of Parkinson's disease have been partially linked as contributing to cellular senescence in dopaminergic neurodegeneration [39]. In VSMCs with CoCl_2_-induced senescence, transcriptional levels of Pbx1 and NRF1 were also reduced.

Epigenetic modifications, including DNA methylation and histone acetylation, were demonstrated to influence gene transcription by changing binding affinities of TFs to their specific cis-regulatory sequence [40, 41]. Some regulatory mechanisms of ROS-dependent epigenetic modifications in aging were also previously documented [42]. Thus, binding levels of TFs on the PDGFR-β promoter should be affected not only by the total amount of active TFs but also by epigenetic changes in the promoter region. However, epigenetic modifications did not affect changes in binding levels in the current experiment because the PCR-amplified promoter sequences had no epigenetic modifications. Hence, differences in TF-binding levels between normal and senescent VSMCs should mainly be affected by the decrease of active TFs in the experiment of TF-binding array (Table 1). Gene quantification results of candidate TFs also supported this argument (Figure 6). So, the suppressions of gene expression and promoter activity of PDGFR-β should also be partially attributed to reductions of active TFs in CoCl_2_-senescent VSMCs (Figures 3A, 4). Although differences in TF-binding levels may mainly be affected by decreases in active TFs based on current data, epigenetic changes and related mechanisms of the PDGFR-β promoter in CoCl_2_-induced senescent VSMCs still need to be clarified in the future.

PDGFRs, transmembrane tyrosine kinase receptors, have two isoforms named PDGFR-α and PDGFR-β, which are activated by the PDGF and then dimerized to trigger signaling pathways involved in cell proliferation, migration, and differentiation [43, 44]. The mitogen-activated protein kinase (MAPK)-ERK1/2 and phosphoinositide 3-kinase (PI3K)-AKT-mTOR axes are two critical downstream signaling pathways of the PDGFR that participate in PDGF-stimulated VSMC proliferation and migration [45]. Numerous evidences have shown that PDGF-BB and PDGFR-β are upregulated in balloon angioplasty-injured arteries and are positively associated with neointimal formation [8, 46, 47]. Thus, PDGFR antagonists have been developed as therapeutic strategies to prevent neointimal hyperplasia [48]. Our data showed that PDGF-BB stimulated activations of the AKT, mTOR, and ERK1/2 proteins, which were markedly decreased in CoCl_2_-induced senescent VSMCs compared to those of normal VSMCs (Figure 3C). These reductions in downstream signaling molecules may be partially attributed to deactivation of the PDGFR-β protein (Figure 3B). Summarizing these experimental results, the expression or activation changes of PDGF-β and its downstream signaling pathways in CoCl_2_-induced senescent VSMCs could provide some pathological mechanisms to support our clinical finding of why the prevalence of restenosis was reduced in the aged population (Figure 7, Table 2).

According to the newest statistical report from the American Heart Association, the prevalence of coronary heart disease (CHD) has an increasing trend with age and shows a significant difference between women and men [49], which suggests that CHD is an age-related disease and its prevalence has a gender difference. Data of this report were separated into four age groups (20~39, 40~59, 60~79, and ≥80 years), and results indicated that the CHD prevalence was positively correlated with age in both genders (0.6%, 6.1%, 19.7%, and 30.6% in males; 0.7%, 5.4%, 11.0%, and 21.7% in females). The female population aged more than 39 years always had a lower prevalence than that in males. Moreover, the difference in prevalence between two genders had a tendency to gradually increase with age, and the largest difference in CHD prevalence between genders was about 8.9% in the oldest group. Cardiovascular protection by female hormones may be part of the possible reason explaining CHD prevention in women [50]. Accordingly, the CHD prevalence should be relatively higher in older or male populations than that in younger or female ones. Our analyzed data also showed a similar trend of >81% of all participants (44 persons) belonging to the older group (>60 years old), and 69.4% of older participants were men (Table 2). To date, the correlation between age and in-stent restenosis is unclear. Our data implied that there was a slight negative correlation between age and the prevalence of restenosis (*r* = -0.057, *p* = 0.725). In addition, our data showed that the female group might have a lower proportion of restenosis than the male group. A previous study also showed a similar finding supporting a gender difference in the prevalence of restenosis, and women presented a lower risk of restenosis after vascular stenting [51]. Estrogen may play a critical protective role in reducing the formation of restenosis by accelerating endothelial cell growth and inhibiting VSMC proliferation and migration [52, 53]. However, our study had few female participants with CHD in the younger group, which could have mainly been due to the following reasons. First, female hormones provide some cardiovascular effects to reduce the probability or prevalence of CHD in younger women. Second, participants with diabetes mellitus, a highly prevalent comorbidity in CHD patients and a major risk factor for in-stent restenosis, were excluded from our study to avoid confounding from diabetes mellitus [54].

The possible relationship between expression of the PDGF receptor and cellular senescence has been explored previously. Aoyagi et al*.* indicated that kinetics of PDGF-BB binding and the expression of beta-subunit of the PDGF receptor were prominently reduced during cellular senescence in human VSMCs from the three strains [55]. Furthermore, tyrosine phosphorylation of the PDGF receptor was also greater higher in young VSMCs than that in aged cells. Besides, it has also been demonstrated that RNA and protein expressions of PDGFR-β were significant diminished in replicative senescent cells and oxidative stress-induced premature senescent cells [56]. In our study, transcriptional, translational and activation levels of PDGFR-β have also been markedly suppressed in oxidative stress-induced senescent VSMCs (Figure 3). PDGF/PDGFR-β signaling pathway plays a critical role in the progression of neointimal hyperplasia (restenosis) through stimulating VSMC proliferation and migration. Thereby, multiple suppressions of PDGFR-β provided partial mechanisms to explain why senescent VSMCs was insensitive to PDGF-BB-mediated stimulation (Figure 2) and prevalence of restenosis was lower in aged population (Table 2). Despite previous studies have collectively linked the causality among senescent VSMCs, PDGFR-β suppression, and prevalence of restenosis in aged arteries. However, in the future, we can still use the rat’s model introduced with balloon angioplasty to further verify the changes of expression profiles in PDGFR-β and its downstream molecules as well as PDGFR-β expression-associated transcription factors within the balloon-injured region of arteries between younger and older rats

Our data from cellular studies suggested that oxidative stress-induced senescent VSMCs are insensitive to stimulation by PDBF-BB, a critical restenosis-promoting factor in cell proliferation and migration. This phenomenon could very possibly help prevent formation of neointimal hyperplasia (or restenosis). Although our clinical study provides some clues that the proportion of restenosis was relatively lower in the older population than in the younger population, the small sample size of eligible participants is a limitation restricting the evaluation of the statistical correlation between age and the prevalence of restenosis. In addition, this study also lacked experimental data from an *in vivo* study, such as an animal aging model, to verify the pathological mechanisms and findings obtained from cellular studies as well as to provide direct and reliable evidence linking the experimental results from the cellular level to the clinic.

Taken together, decreases in binding levels and gene expressions of some TFs might play critical roles resulting in suppression of the PDGFR-β gene in senescent VSMCs. Subsequently, a desensitization phenomenon of senescent VSMCs to PDGF-BB-stimulated cell proliferation and migration occurred. Although our clinical data indicated that the older population had a relatively low proportion of in-stent restenosis compared to the younger population, more participants and *in vivo* experiments are still needed to clarify the relationship between age and the prevalence of restenosis.

## MATERIALS AND METHODS

### Chemicals and reagents

Dulbecco’s modified Eagle medium (DMEM; #12800-017) was purchased from Gibco (Rockville, MD, USA). Hyclone™ Antibiotic Antimycotic solution (#SV30079.01) and fetal bovine serum (FBS; FetalClone III™, #SH30109.03) were procured from GE Healthcare Life Sciences (Chicago, IL, USA). Primary antibodies against phospho-AKT (#GTX50128), PDGFR-β (#GTX61115), phospho-PDGFR-β (#GTX61797), and HIF-1α (#GTX127309), as well as horseradish peroxidase (HRP)-conjugated secondary antibodies against mouse (#GTX213112-01) and rabbit (#GTX213110-01) immunoglobulin G (IgG) were purchased from GeneTex (Irvine, CA, USA). Antibodies detecting β-actin (#66009-1-Ig), phospho-extracellular signal-regulated kinase 1/2 (ERK1/2; #05-797R), and phospho-mammalian target of rapamycin (mTOR; #ab109268) were respectively obtained from Proteintech (Rosemont, IL, USA), Millipore (Bedford, MA, USA), and Abcam (Cambridge, MA, USA). Recombinant human PDGF-BB (#100-14B) and AG-1295 (#14529), a PDGFR-β inhibitor, were respectively bought from PeproTech (Rocky Hill, NJ, USA) and Cayman Chemical (Ann Arbor, MI, USA). Cobalt dichloride (#C8661) was obtained from Sigma-Aldrich (St. Louis, MO, USA).

### Cell culture

A10 cells (#60082), a cell line of rat thoracic aorta smooth muscle, were obtained from the Bioresource Collection and Research Center (Hsinchu, Taiwan) and maintained in DMEM supplemented with 10% (v/v) FBS and a 100x-diluted antibiotic antimycotic solution. Cells were kept at 37°C in a humidified incubator with 5% (v/v) CO_2_ and 95% (v/v) air. The culture medium was refreshed every 2~3 days.

### Induction and identification of cellular senescence

Cellular senescence of A10 cells was induced by 72 hr of incubation with cobalt dichloride at a concentration of 150 or 300 μM, and was identified by detecting intracellular senescence-associated beta-galactosidase (SA-β-gal) activity according to previous reports [57, 58]. Briefly, cells were washed twice with 1× phosphate-buffered saline (PBS), fixed with a 2% (v/v) glutaraldehyde-PBS solution for 5 min at room temperature, washed three times in PBS for 1 min each, and then stained with a staining solution containing 40 mM of citric acid/Na phosphate buffer (pH 6.0), 5 mM of K_4_[Fe(CN)_6_]·3H_2_O, 5 mM K_3_[Fe(CN)_6_], 150 mM sodium chloride, 2 mM magnesium chloride, and 1 mg/mL X-gal. After overnight incubation at 37°C, cells were washed twice with PBS for 30 s each, rinsed with methanol, and air-dried. Subsequently, cells were photographed using ToupView™ image acquisition software (ToupTek Photonics, Zhejiang, China) at 40× magnification under an inverted microscope (#Eclipse TS100; Nikon, Melville, NY, USA) equipped with a digital camera (#E3ISPM06300KPA-IP106300A; Suzhou Vision Photonics, Jiangsu, China).

### Cell growth analysis by a 3-[4,5-dimethyl thiazol-2-yl]- 2,5-diphenyl tetrazolium bromide (MTT) assay

These experiments were carried out according to our previous study [59]. Briefly, cells (10^4^ cells/well) were seeded on a 96-well plate for 24 hr of adaptation. After that, the culture medium was refreshed with serum-free medium with or without 25 ng/mL PDGF-BB. After 24 hr of incubation, cells were treated with 5 mg/mL of MTT for 2 hr. Subsequently, cells were washed with PBS, and then 100 μL of dimethyl sulfoxide (DMSO) was added to each well. Absorbance values at 570 nm were determined for each well using 650 nm as the reference wavelength. Cell growth is presented as the ratio (multiple of change) of the absorbance of cells treated with PDGF-BB to that without PDGF-BB treatment.

### Wound-healing assay

This experimental procedure was according to a method described in our previous study [60]. Briefly, cells were seeded on 12-well plates (5 × 10^5^ cells/well). After 24 hr of serum-free starvation, a pipette tip was used to create an original cell-free region on the confluent cell monolayer, which was then photographed at 0 hr under a microscope at 40× magnification. After that, cells were treated with 6 ng/mL of PDGF-BB. At 16 hr after treatment, the cell number within the original cell-free region was counted on photographic images using the particle analysis function of Image J analytical software (National Institutes of Health (NIH), Bethesda, MD, USA) according to the official web-based manual (https://imagej.net/Particle_Analysis). The extent of cell migration is shown as the multiple of change of migrating cell numbers compared to the normal cell group.

### Immunoblotting

Immunoblot experiments were carried out as described previously [59]. Harvested cells were lysed using lysis buffer containing 25 mM Tris-HCl (pH 7.6), 150 mM NaCl, 0.1% (w/v) sodium dodecylsulfate (SDS), 5 mM ethylenediaminetetraacetic acid (EDTA; pH 8.0), 1 mM dithiothreitol (DTT), 1% (v/v) Triton X-100, 20% (v/v) glycerol, a proteinase inhibitor cocktail (#4693159001; cOmplete™; Roche, Basel, Switzerland), and a phosphatase inhibitor cocktail (#04906837001; PhosSTOP™; Roche). Cell lysates were further centrifuged at 13,000 ×*g* and 4 °C for 10 min to collect the supernatants for SDS-polyacrylamide gel electrophoresis (PAGE). Protein concentrations were measured with a Bio-Rad protein assay kit (Bio-Rad, Hercules, CA, USA) according to the manufacturer’s instructions. Aliquots containing 30 μg of protein were electrophoresed using 10% slab SDS-PAGE gels and then transferred to polyvinylidene difluoride membranes (Immun-bot^®^; Bio-Rad). After blocking non-specific binding sites with 5% (w/v) non-fat milk at room temperature for 1 h, the membrane was incubated with primary antibodies overnight at 4°C, followed by HRP-conjugated secondary antibodies at 4°C for 3 hr. Substrates were visualized using a T-Pro LumiLong Plus Chemiluminescent Substrate Kit (T-Pro Biotechnology, New Taipei City, Taiwan). The luminescence signal was acquired by the Azure C300 imaging System (Azure Biosystems, Dublin, CA, USA) and quantified using AzureSpot software (v14.0; Azure Biosystems). Results for each experiment were normalized to the band density of β-actin. The relative protein expression of the group without CoCl_2_ treatment was defined as 100%.

### Analysis of gene expressions

The procedures examining gene expressions were conducted according to our previous study [61]. Briefly, 1 mL of 3-Zol™ reagent (#2001; MDBio, Taipei, Taiwan) was added to harvested cells, which were then vortexed for 30 s and incubated on ice for 5 min. After that, 0.2 mL of chloroform was added to the cell lysate, vortex-mixed for 15 s, and incubated at room temperature for 3 min. After centrifugation at 12,000 ×*g* for 15 min, the aqueous phase was transferred to a clean tube, precipitated with 0.5 mL of isopropanol, and centrifuged at 12,000 ×*g* for 15 min. The pellet was then washed with 1 ml of 75% (v/v) cold ethanol prepared with 0.1% (v/v) diethyl pyrocarbonate (DEPC)-treated water, centrifuged at 12,000 ×*g* at 4°C for 15 min, dried for 20 min at room temperature, re-suspended in 50 μL DEPC-treated water, and stored at -80°C.

Complementary (c)DNA synthesis was carried out using the ReverTra Ace set (#PU-TRT-200; TOYOBO, Osaka, Japan) according to the manufacturer’s manual. Briefly, 1 μg of total RNA was supplemented in a total reaction volume of 20 μL with 1× reverse-transcription buffer, 1 mM dNTPs, 0.5 nM oligo(dT)20, 0.5 units of an RNase inhibitor, and 5 units of ReverTra Ace (reverse transcriptase). After incubation for 20 min at 42°C, the mixture was incubated for 5 min at 99°C to denature the products. Finally, the cDNA product was stored at -80°C.

A traditional polymerase chain reaction (PCR) was conducted using the OnePCR™ HotStar system (#SM206-0100; GeneDireX, Miaoli, Taiwan) in a thermocycler (Labcycler™; SensoQuest, Gottingen, Germany). Briefly, the reaction mixture contained 1 μg cDNA, 0.2 μM primer (Table 3), and 25 μL of the OnePCR™ HotStar reagent in a total volume of 50 μL. After hot-start activation for 5 min at 94°C, 20~35 cycles were carried out, each consisting of 30 s at 94°C, 1 min at 60°C, and 2 min at 72 °C. PCR products were electrophoresed on a 2% agarose gel in 0.5× Tris-borate-EDTA (TBE) running buffer at 100 V for 1 h. DNA bands were visualized with a fluorescent dye (Novel Juice™; GeneDireX, Taichung, Taiwan). The band signals were acquired and quantified with the Azure C300 imaging System and AzureSpot software (Azure Biosystems), respectively. The band intensity of PDGFR-β was normalized to that of the internal control (GAPDH), and the relative gene expression of the group without CoCl_2_ treatment was defined as 100%.

**Table 3:** Information of PCR primers.

**No.**	**Gene name (accession no.)**	**Sequences of primer pairs**
1	**COUP-TF** (NM_080778.2)	CCAACCGGAACTGTCCCATC	TGCAAACTGCCCGTGAGTAG
2	**FOXD3** (XM_008763960.1)	GGGCAAGGGTAACTACTGGAC	TAGGCTCCGAAGCTCTGCATC
3	**GAPDH** (NM_017008.4)	ATCAAGAAGGTGGTGAAGCAGGCG	GGGATGGAATTGTGAGGGAGATGCTC
4	**NFATc1** (NM_001244933.1)	CAGCTACCCGGTCATTGGAG	CTTGCACAGGTCTCGGTCAG
5	**NFATc2** (NM_001107805.1)	CAGTCAAACAGGAGCAGAACC	AAGGCGTCGTGCGATACTG
6	**NFATc3** (XM_008772519.1)	GTGGCCATCCTGTTGTGAAG	TCCAGTAATGCGATGCACCTG
7	**NFATc4** (NM_001107264.1)	GGATCCAACTTCCTGCCAGAC	GGGATGGTCAGAGTCAGTGTC
8	**NRF1** (NM_001100708)	CCGTTGGAGCACTTACTGGAG	CATTACTTCCGCCATAATGAATCCC
9	**OCT4** (NM_001009178)	GTGAAGTTGGAGAAGGTGGAAC	GTGAAGTTGGAGAAGGTGGAAC
10	**Pbx1** (NM_001134862.1)	GAAGTGCGGCATCACAGTCTC	TTCCATGGGCTGACACATTGG
11	**PDGFR-β** (XM_006254789)	ATCCCAGATACACCCCACGATG	TCCTTACTCCCCAGACACTTGC
12	**PDGFR-β promoter** (2 kb; -2001 ~ -1)	AGACTCGAGTGGGACTGGAGAAGAGGAAGG	AAAGAGATCTGGGCCGATTCTGATTGGCCAAGCTTG
13	**PDGFR-β promoter** (1.5 kb; -1526 ~ -1)	GGCACTCGAGTGGGTGACCTCGGGCAATC	AAAGAGATCTGGGCCGATTCTGATTGGCCAAGCTTG
14	**PDGFR-β promoter** (1 kb; -1021 ~ -1)	GCTTCTCGAGCTTGCTGCTTCTGGAGTCTAAGAATAC	AAAGAGATCTGGGCCGATTCTGATTGGCCAAGCTTG
15	**PDGFR-β promoter** (0.5 kb; -523 ~ -1)	ACTACTCGAGGTATCAGCGCTTCGTTTACAGATG	AAAGAGATCTGGGCCGATTCTGATTGGCCAAGCTTG
16	**PPAR-α** (NM_013196)	GCGAGCCAAGACTGAAGTTC	TCTGCTTCAAGTGGGGAGAG
17	**PPAR-β** (AJ306400)	TATCCGCAAGCCCTTCAGTG	GCAAGGTCTCACTCTCCGTC
18	**PPAR-γ** (NM_013124)	AGATCCTCCTGTTGACCCAGAG	CCACAGAGCTGATTCCGAAG
19	**PXR** (NM_052980.2)	CCCTCACCCTTCAAAGTGGAC	CATGGTTCCACCTCTCCTCAG
20	**SATB1** (NM_001012129)	ATACAATTTCAGGGGAAGTCGC	CAGATCACCTGCCAGAACAC
21	**STAT5** (NM_017064.1)	GGCTCACTACAACATGTACCCA	AGCGTTCAGGACAAGGAGCTT
22	**TFIID** (NM_001004198)	ACCGTACATCTCAGCTGCTTC	ATCGTCACGCACCATGAAAC
23	**USF-1** (NM_031777.2)	GGAAATTGGCGACTGAAGCG	CTGTCCCCTCTTCGGTTTCG

A real-time PCR was performed using the Smart Quant Green Master Mix with dUTP and ROX (#SA-SQGR-V2-1ml; Protech, Taipei, Taiwan) in an ABI Prism 7300 sequence detector (Applied Biosystems, Foster City, CA, USA) according to the procedure of our previous study with minor modifications [61]. The reaction mixture contained 1 μg cDNA, 2 μL of each primer (10 μM), and 10 μL of the Master Mix in a total volume of 20 μL. After hot-start activation for 15 min at 95°C, 40 cycles were carried out, each consisting of 15 s at 95°C, 15 s at 59°C, and 30 s at 72°C. The primer pairs used for the real-time PCR are listed in Table 3. The relative transcript expression was calculated using the equation 2^–ΔΔCt^, and results are presented as multiples of change relative to the control group.

### Plasmid construction and promoter activity assay

The sequence of the potential promoter region (ranging -2001 to -1) of the rat PDGFR-β was identified by aligning sequences of PDGFR-β messenger (m)RNA (#XM_006254789) and a chromosome 18 fragment (#NC_005117) obtained from the GenBank genetic sequence database. Four promoter segments of different lengths, of 2001, 1526, 1021, and 523 bp, were individually amplified by a PCR using appropriate primer pairs with restriction enzyme recognition sequences (Table 3). Amplified promoter segments were further cloned into the pGL4.10[*Luc2*] plasmid (#E6651; Promega, Madison, WI, USA) using digestion of the restriction enzymes, *Xho*I (#R6161; Promega) and *Bgl*II (#R6081; Promega), to construct four complete reporter plasmids, pGL4.10[*Luc2*]-rPDGFR-β, with different PDGFR-β promoter regions. The promoter sequence within the recombinant plasmids was verified with Sanger sequencing conducted by Mission Biotech (Taipei, Taiwan).

Cells seeded on 6-well plates (at ca. 80% cell confluence) were transfected with the four individual pGL4.10[*Luc2*]-rPDGFR-β plasmids using the TransIT^®^-LT1 transfection reagent (#2300; Mirus Bio LLC, Madison, WI, USA) according to the procedure in the manufacturer’s manual. In brief, 2.5 μg of plasmid DNA was mixed with 250 μL serum-free culture medium and then mixed with 7.5 μL of transfection reagent for a 30-min incubation at room temperature to form a stable complex of plasmid DNA/transfection reagent. After that, medium within the wells was refreshed with 2.5 mL of culture medium, and the mixture of plasmid DNA/transfection reagent complex was added drop-wise to different areas of the wells. After a 24-h incubation for cell transfection, PDGFR-β promoter activity was measured with a commercial luciferase assay system kit (#E4030; Promega) in accordance with the manufacturer’s instructions. Briefly, cells were washed twice with PBS, and then 200 μL of 1× Reporter lysis buffer was added to each well. After a 15-min incubation at -20°C, cell lysates were harvested, vortexed for 15 s, and then centrifuged at 13,000 rpm for 5 min at 4°C. After that, 20 μL of supernatant was mixed with 100 μL of luciferase assay reagent. The luminescent signal (or relative light units; RLU) of cell supernatants was detected with a luminometer (Varioskan™ Flash; Thermo Scientific, Waltham, MA, USA) with the following parameters: a 2-s measurement delay followed by a 10-s measurement read. The RLU value was further normalized to the protein concentration from the same cell supernatant. Finally, the normalized RLU value of the pGL4.10[*Luc2*]-rPDGFR-β plasmid with the 0.5-kb promoter segment was defined as 100%.

### Analysis of a TF-binding array

Nuclear proteins were extracted using the Nuclear Extraction Kit (#SK-0001; Signosis, Santa Clara, CA, USA) according to the manufacturer’s instructions. A rat PDGFR-β promoter segment (523 bp; -523 to -1) was amplified using a PCR, and PCR clean-up was conducted using a Plus DNA clean/Extraction kit (#DP034P; GMbiolab, Taichung, Taiwan). After that, binding profiles of 96 TFs on the rat’s PDGFR-β promoter segment were examined using Promoter-Binding TF Profiling Plate Array II (#FA-2002; Signosis). Briefly, 8 μg of the nuclear extract, 0.3 μM of the PDGFR-β promoter segment, and a competitive biotin-labeled TF oligo probe were well mixed and then incubated in the TF binding buffer mixed solution for 30 min at room temperature. Subsequently, the TF-bound oligo probes were separated using a membrane-based spin column (Isolation column) and then hybridized with complementary sequences labeled in different wells of a 96-well plate. After that, the captured oligo probes were detected with streptavidin-HRP and a chemiluminescent substrate. Luminescent signals expressed as relative light unit (RLU) were quantified with a luminometer, and RLU values of <200 were considered background. Final data are presented as the ratio of the RLU value in normal cells to that in senescent cells.

### Bioinformatics prediction of binding motifs

The rat PDGFR-β promoter sequence (ranging -523 to -1) was analyzed using web-based bioinformatics tools (PROMO and MatInspector^®^) for predicting putative TF-binding sites (TFBSs) defined in the TRANSFAC^®^ database [62–64]. In the promoter analysis with PROMO (ALGGEN, Barcelona, Spain), parameters were set up by restricting both factor species and site species as animals with a maximum matrix dissimilarity rate of ≤5%. In addition, putative TFBSs within this promoter segment were also analyzed by MatInspector (Genomatix AG, Munich, Germany) using default parameters.

### Collection of clinical data

Clinical studies (#201405019 and #N201603042) were approved by the Taipei Medical University (TMU) Joint Institutional Review Board (TMU-JIRB) and were performed from June 2015 to June 2017. In total, 104 patients were recruited from TMU Hospital, Wan-Fang Hospital, and Shuang-Ho Hospital in Taiwan. Inclusion criteria were patients (≥30 years old) who had received vascular stenting, and patients suffering from diabetes, hepatitis, uremia, cancer, hemophilia, or autoimmune diseases were excluded. Restenosis was defined as >50% diameter stenosis within the vascular lesion with vascular stenting, and the vascular status (with or without restenosis) was diagnosed at the first follow-up visit (6 months after surgery). Finally, the medical records of 44 eligible patients were collected to analyze the relationship between age and vascular status (with or without restenosis). At the end of the clinical studies, 60 patients who had been diagnosed with diabetes, a common comorbidity of restenosis, or who had withdrawn from the study were excluded.

### Statistical analysis

All data are presented as the mean ± standard deviation (SD). The experiments were run in triplicate. Statistical significance among multiple groups was evaluated by a one-way analysis of variance (ANOVA). Pearson's correlation test was used to analyze the correlation between age and vascular status (with or without restenosis) of patients who had been followed-up 6 months after vascular stenting. A value of *p* < 0.05 was regarded as statistically significant.
